# Metabolic Profiling Early Post-Allogeneic Haematopoietic Cell Transplantation in the Context of CMV Infection

**DOI:** 10.3390/metabo13090968

**Published:** 2023-08-22

**Authors:** Kirstine K. Rasmussen, Quenia dos Santos, Cameron Ross MacPherson, Adrian G. Zucco, Lars Klingen Gjærde, Emma E. Ilett, Isabelle Lodding, Marie Helleberg, Jens D. Lundgren, Susanne D. Nielsen, Susanne Brix, Henrik Sengeløv, Daniel D. Murray

**Affiliations:** 1 Centre of Excellence for Health, Immunity and Infections (CHIP), Rigshospitalet, Copenhagen University Hospital, 2100 Copenhagen, Denmark; 2 Bone Marrow Transplant Unit, Department of Hematology, Rigshospitalet, Copenhagen University Hospital, 2100 Copenhagen, Denmark; 3 Center for Basic Metabolic Research (CBMR), Copenhagen University, Blegdamsvej 3, 2200 Copenhagen, Denmark; 4 Department of Infectious Diseases, Rigshospitalet, Copenhagen University Hospital, 2100 Copenhagen, Denmark; 5 DTU Bioengineering, Department of Biotechnology and Biomedicine, Technical University of Denmark, 2800 Kgs. Lyngby, Denmark

**Keywords:** aHSCT, WGCNA, metabolomics, lipidomics, cytomegalovirus, CMV, correlation network analysis, TMAO

## Abstract

Immune dysfunction resulting from allogeneic haematopoietic stem cell transplantation (aHSCT) predisposes one to an elevated risk of cytomegalovirus (CMV) infection. Changes in metabolism have been associated with adverse outcomes, and in this study, we explored the associations between metabolic profiles and post-transplantation CMV infection using plasma samples collected 7–33 days after aHSCT. We included 68 aHSCT recipients from Rigshospitalet, Denmark, 50% of whom experienced CMV infection between days 34–100 post-transplantation. First, we investigated whether 12 metabolites selected based on the literature were associated with an increased risk of post-transplantation CMV infection. Second, we conducted an exploratory network-based analysis of the complete metabolic and lipidomic profiles in relation to clinical phenotypes and biological pathways. Lower levels of trimethylamine N-oxide were associated with subsequent CMV infection (multivariable logistic regression: OR = 0.63; 95% CI = [0.41; 0.87]; *p* = 0.01). Explorative analysis revealed 12 clusters of metabolites or lipids, among which one was predictive of CMV infection, and the others were associated with conditioning regimens, age upon aHSCT, CMV serostatus, and/or sex. Our results provide evidence for an association between the metabolome and CMV infection post-aHSCT that is independent of known risk factors.

## 1. Introduction

Allogeneic haematopoietic stem cell transplantation (aHSCT) is a potentially curative treatment performed to restore normal haematopoiesis and/or treat haematological malignancies [1]. aHSCT is associated with severe immune disruption due to the preceding conditioning regimen and immune suppressive therapy, and the subsequent reconstitution of the donor immune system [2]. This disruption predisposes the transplant recipients to a high risk of developing severe infections, including cytomegalovirus (CMV) infection. The current prophylaxis strategies have decreased the number of post-transplantation CMV infections [3]; however, CMV disease continues to cause morbidity and mortality [4,5,6,7]. CMV infection can cause fever, cytopenia, allograft rejection, and, in severe cases, tissue-invasive disease [7,8]. Therefore, the identification of high-risk patients is crucial to improve patient outcomes. A variety of factors have been identified relating to the risk of CMV infection, e.g., donor/recipient serotype mismatch, a myeloablative conditioning regimen, HLA-mismatched/unrelated donors, graft-versus-host disease (GvHD), and underlying disease [1,5,8,9]. However, these factors cannot fully explain or predict all cases of CMV infection. Newer technologies, such as the Quantiferon^®^-CMV assay, have improved risk stratification but are still unable to explain all cases [10]. This indicates that there remains a gap in our understanding of the underlying biological mechanisms that explain why some patients acquire a CMV infection while others do not. Metabolites have previously been described as proximal reporters of disease [11,12] and, therefore, may reveal crucial information about the host/pathogen interactions relating to CMV infections in aHSCT recipients.

Several studies have identified metabolites that are potentially relevant for CMV infection. These studies include findings pointing to a glutamine requirement for viral replication [13] and associations between multiple metabolites and lipids with CMV infection in kidney transplant recipients [14] and aHSCT recipients [15]. However, these prior studies were either performed in cell lines or in relatively small clinical cohorts, and their results have not been validated in other cohorts. Another major limitation of these studies is that they focused on single-metabolite associations. Metabolites and lipids are known to perform their biological functions as part of molecular pathways, and the study of individual metabolites may not fully elucidate underlying metabolic disruptions [12].

In this study, we first investigated whether a set of selected metabolites that had previously been associated with CMV infection were associated with a risk of subsequent CMV infection early post-aHSCT in a well-described clinical cohort [16,17,18]. Additionally, we sought to establish a framework for a pathway-resolved, network-based discovery analysis to explore the complete metabolic disruptions early post-aHSCT using untargeted metabolomics and lipidomics data. This process included the unsupervised clustering of metabolites and lipids that may share biological functions and the investigation of their relation to subsequent CMV infection or clinical traits associated with CMV infection.

## 2. Materials and Methods

### 2.1. Patient and Sample Selection

The patients included in this study underwent aHSCT at the Stem Cell Transplantation Unit, Department of Hematology, Rigshospitalet, Copenhagen University Hospital, Denmark, from January 2016 to October 2017. Only patients with at least one plasma sample taken within 7–33 days post-transplantation were considered. Cases were patients who were infected with CMV within 34–100 days post-aHSCT. CMV infection was defined as either two positive CMV-PCR tests (viral load ≥ 273 IU/mL) with a maximum of 14 days between tests or a single positive CMV-PCR test (viral load ≥ 2730 IU/mL) [3]. Controls were aHSCT patients who did not acquire a viral infection within 100 days post-aHSCT. Cases and controls were matched according to sex, conditioning regimen, and CMV risk score (combined CMV IgG serostatus of donor (D) and recipient (R) at the time of transplantation). Cases that could not be matched according to CMV risk score were matched according to sex and conditioning regimen only.

### 2.2. Sample Selection

Plasma samples were sourced from the Personalised Medicine for Infectious Complications in Immune Deficiency (PERSIMUNE) biobank and the Department of Clinical Microbiology, Rigshospitalet biobank (both of which have been approved by the Danish Data Protection Agency, respectively; RH201504, I-Suite 03605 and RH2016194, I-Suite 04769). If patients had more than one plasma sample collected within 33 days post-aHSCT, the latest collected sample was selected.

### 2.3. Clinical Data

Clinical traits and patient outcomes were retrieved from the PERSIMUNE Data Warehouse and from a clinical database at the Department of Hematology, Rigshospitalet. These data included conditioning regimen (myeloablative or non-myeloablative), donor source, and CMV risk scores (high risk = D−/R+, intermediate risk = D+/R+, and low risk = D+/R− and D−/R−). Patients with an unknown CMV risk score were assigned to the low-risk group as they presented a similar incidence risk for CMV infection upon conducting a univariable analysis (Table S1).

### 2.4. Conditioning Regimens

All patients in the cohort underwent either myeloablative (MAC; myeloablative conditioning) or non-myeloablative (Mini) conditioning prior to aHSCT. The main MAC regimen included total body irradiation (TBI) with 1200 cGy combined with cyclophosphamide or a combination of fludarabine/treosulfan. The main Mini regimen included TBI with ≤400 cGy and fludarabine.

### 2.5. CMV Monitoring and Treatment

All transplant recipients were monitored weekly using CMV-PCR tests concerning plasma from days 21–150 post-aHSCT. Following a positive CMV-PCR result, pre-emptive treatment with valganciclovir was initiated, which entailed the administration of 900 mg of valganciclovir twice a day for at least two weeks and until a negative CMV-PCR result was obtained. Following the onset of CMV disease (i.e., CMV infection with symptoms), treatment with ganciclovir was initiated, which entailed the administration of 5 mg/kg of ganciclovir twice a day for 10–14 days followed by 5 mg/kg once a day for at least 5–7 days and until the patient no longer exhibited symptoms.

### 2.6. Metabolomics and Lipidomics Analysis

Plasma samples were analysed by Metabolon Inc. (Morrisville, NC, USA) [19] using untargeted ultra high-performance liquid chromatography/tandem accuracy mass spectrometry (UHPLC-MS/MS) methods. Metabolites were detected as relative concentrations and were annotated using the Metabolon reference library. Metabolite peaks that were not matched with the database were included in the dataset with unique random IDs. Lipids were detected as absolute concentrations using single-point quantification and the Metabolon Complex Lipid Panel™. All known metabolites and lipids were assigned a superpathway (top-level classification of biochemical reactions related to the metabolism of similar compounds) and a subpathway (lower-level pathway category nested within superpathways).

### 2.7. Pre-Processing of Metabolomics and Lipidomics Data

Metabolite and lipid abundances were median-scaled relative to the individual molecule across all samples (this process was performed by Metabolon). Molecules with low variance were removed from each dataset by using the *nearZeroVar* function from the *caret* R package [20] (frequency cut = 95/5; unique cut = 10%). Before conducting the exploratory analysis of metabolomic and lipidomic profiles, the datasets were additionally log2-transformed to ensure a normal distribution (Figure S1).

### 2.8. Statistical Analysis

Statistical differences between demographic and clinical traits of the cases and controls were assessed using Chi-square test for categorical variables, Wilcoxon Independent Rank-Sum test for continuous variables, and Fisher’s exact test for categorical variables with ≤5 patients in at least one category.

As this was a case–control study with a binary outcome variable (CMV infection or no CMV infection), we used multivariable logistic regression models to test associations between selected metabolites previously associated with CMV infection (Table S2) and subsequent CMV infection in this cohort. Models were adjusted for sex, age upon aHSCT, conditioning regimen, and CMV risk score. The following metabolites were included: alanine, choline, glutamine, kynurenine, lactate, lysine, phenylalanine, quinolinate, taurine, total free fatty acids (FFA), trimethylamine N-oxide (TMAO), and tryptophan. Results were reported using odds ratios, confidence intervals, and unadjusted *p*-values using a significance threshold of *p* < 0.05.

### 2.9. Pathway-Resolved Correlation Network Analysis

Pathway-resolved correlation-network-based investigation of metabolic and lipidomic profiles early post-aHSCT was performed for each dataset individually using the Weighted Gene Co-expression Network Analysis (*WGCNA*) R package [21]. Signed co-abundance networks were constructed using the bi-weight mid-correlation measure as suggested by Pedersen et al. 2018 [22]. Weighted network adjacencies ($a_{i,j}$) were calculated as defined by Langfelder and Horvath 2008 [21] using the following equation:

(1) $$a_{i,j}={\left|\frac{1+cor(x_{i},x_{j})}{2}\right|}^{\beta },\;where\;\beta >1$$

Here, $cor(x_{i},x_{j})$ is the correlation between metabolites $x_{i}$ and $x_{j}$, and β is the soft-threshold power selected for each dataset by fitting the data to a scale-free topology model. Using this method, we can model the structure of biological data for which the network node degree usually follows an exponential curve rather than being a constant. Further, the method allows the emphasis of strong correlations in the network. For the metabolomics data, a power of β = 16 was selected, and for the lipidomics data, a power of β = 30 was selected (power plots are shown in Figure S2). From the adjacency matrix, a topological overlap matrix was created using the following equation, as described by Langfelder and Horvath 2008 [21]:

(2) $$TOM\left(x_{i,j}\right)=\frac{a_{i,j}+\sum_{k\neq i,j}a_{i,k}a_{k,j}}{\min\left(k_{i},k_{j}\right)+1-a_{i,j}}$$

Here, the parameters $k_{i}$ and $k_{j}$ represent the connectivity for each of the molecules *i* and *j*. Detection of modules (clusters of highly interconnected metabolites/lipids measured according to the topological overlap) was performed using hierarchical clustering with average linkage and the Dynamic Tree Cut method. The minimum number of metabolites/lipids required to form a separate module was determined by performing module detection for minimum module sizes of 5–20 (visual workflow is shown in Figure S3). The minimum module size value resulting in a reasonably low number of modules while maintaining high correlation within modules (module membership) was selected. A minimum module size of 10 was selected for the metabolomics dataset, while 12 was selected for the lipidomics dataset (iteration outputs are shown in Table S3). Final modules were tested with respect to correlations with clinical traits relating to CMV infection (sex, age, conditioning regimen, and CMV risk score) using Spearman’s Rank correlation evaluated using Benjamini–Hochberg adjusted *p*-values (q-values) and a significance level of q < 0.05. The metabolic pathway content was assessed at super- and subpathway levels using annotations provided by Metabolon. Unannotated metabolites were included as unknowns.

### 2.10. Software

All statistical and bioinformatics analyses were performed in R version 3.6.3 [23]_,_ and all code is available in GitHub (https://github.com/PERSIMUNE/PAC2022Rasmussen_CMV_metabolomic_profiling (accessed on 22 December 2022)).

## 3. Results

### 3.1. aHSCT Patient Cohort

The cohort consisted of 68 aHSCT patients, 34 of which (50%) acquired a CMV infection between days 34–100 post-transplantation (cases), while the other 34 (50%) did not have a positive CMV-PCR result within 100 days post-transplantation (controls). Nineteen cases were matched by criteria that includeda CMV risk score, while fifteen were matched only by sex and conditioning regimen. The cohort’s characteristics are shown in Table 1, and the sample collection times are presented in Figure S4. There were no differences between the groups with regard to sex, age at the time of transplantation, sample collection time, graft origin, or conditioning regimen. The distributions of CMV risk scores differed between cases and controls, with higher proportions of high-risk constellations amongst the cases (*p* < 0.001) (Table 1).

### 3.2. Metabolomics and Lipidomics Data

UHPLC-MS/MS analysis detected 976 metabolites from 9 superpathways and 34 subpathways and 975 lipids from 14 superpathways and 102 subpathways. During pre-processing, 54 metabolites and 42 lipids were removed from the datasets due to their low variance. Final datasets included 922 metabolites and 933 lipids (the complete list is included in the Supplementary HTML file). Distributions before and after log2-transformation are shown in Figure S1.

### 3.3. Single-Marker Associations with CMV Infection

The multivariable logistic regression models showed an association between trimethylamine N-oxide (TMAO) and subsequent CMV infection (OR = 0.63, 95% CI = [0.41; 0.87], *p* = 0.01) regardless of sex, age at the time of aHSCT, conditioning regimen, and CMV risk score (all results are shown in Table S4). None of the other selected metabolites were associated with CMV infection.

### 3.4. WGCNA Module Associations with Clinical Traits

**Figure 1:**
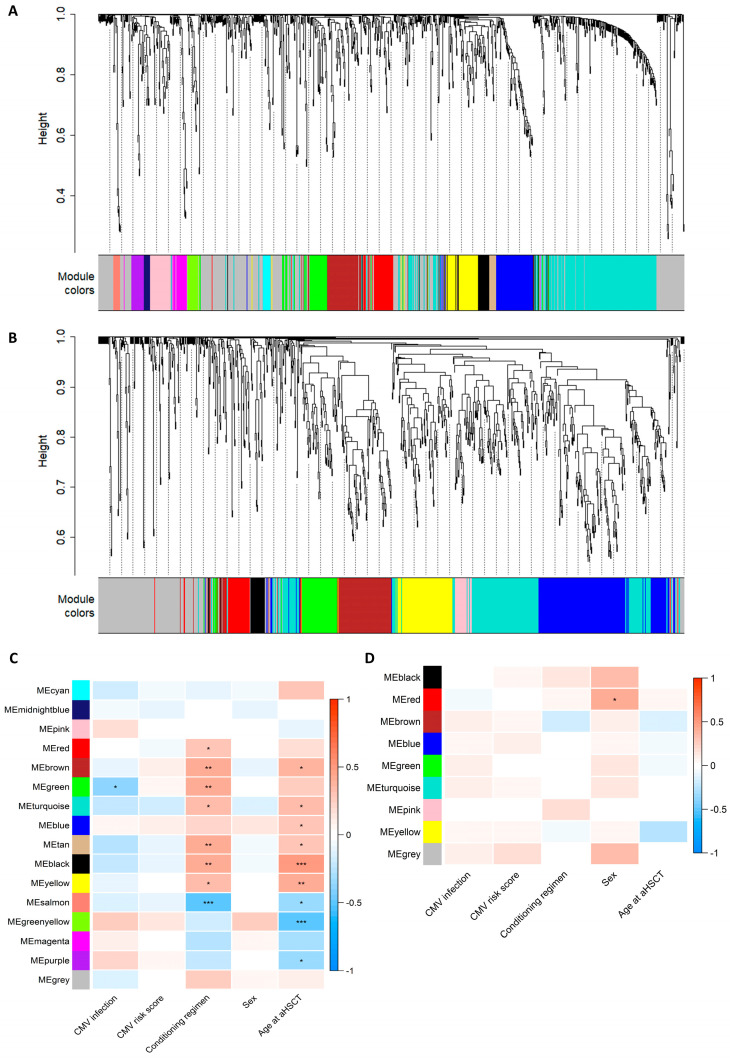
Assessment of modules constructed using the weighted correlation analysis of the metabolomics or lipidomics data. (**A**,**B**) Dendrogram showing hierarchical clustering of the metabolites (**A**) or lipids (**B**), where distance measured in topological overlap is shown on the *y*-axis. The colour bar below indicates which module (cluster) each metabolite/lipid was placed within. The grey module serves as a bin for molecules that do not fit in any other module. (**C**,**D**) Spearman’s rank correlation heatmap of module eigengenes (MEs) for each module (*y*-axis) and clinical traits (*x*-axis). Significance indicated by Benjamini–Hochberg-adjusted *p*-values: * q > 0.05, ** q > 0.01, and *** q > 0.001. CMV infection: 0 = no CMV and 1 = CMV; CMV risk score: 1 = low risk (D+/R− and D−/R−), 2 = intermediate risk (D+/R+), and 3 = high risk (D−/R+); conditioning regimen: 1 = myeloablative (MAC) and 2 = non-myeloablative (Mini); sex: 1 = male and 2 = female; age upon aHSCT: integer (17–73). An interactive version of the heatmaps can be found in the Supplementary HTML file.

**Figure 2:**
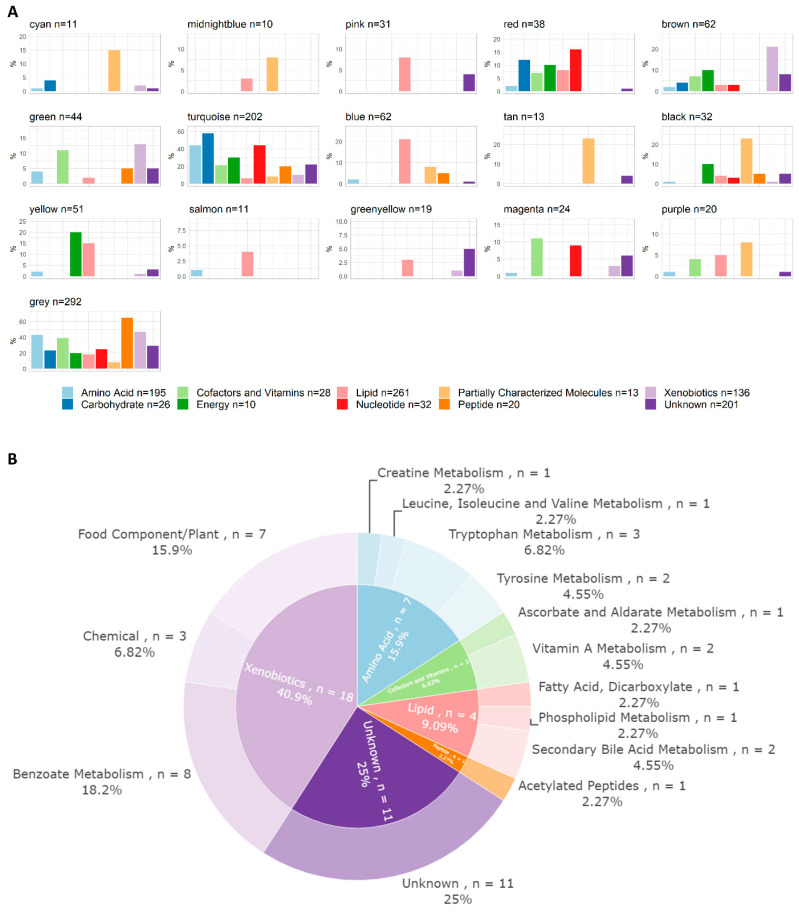
Pathway content of metabolite modules. (**A**) Pathway annotations of metabolites per module at the superpathway level. Superpathways are represented on the *x*-axis by color-coded bars. The percentage of molecules annotated to each superpathway placed in each module is indicated on the *y*-axis; for example, 15% of all acetylated peptides are situated within the cyan module. (**B**) Pathway content of the green metabolite module (*n* = 44), which was found to be inversely correlated with CMV infection. Annotations were derived from the files supplied by Metabolon. The inner circle represents the superpathway level (same colour-coded categories as in (**A**)) and the outer one denotes the subpathway level. An interactive version of this figure, and the corresponding figures of all other modules, can be found in the Supplementary HTML file.

The correlation network analysis resulted in 16 metabolite and 9 lipid modules ranging in size from 10 to 292 molecules (Figure 1A,B and Table S5). The module membership distributions for all the modules are shown in Figure S5. Hierarchical clustering dendrograms and correlation heatmaps for the metabolite and lipid module eigengenes are shown in Figure S6. One metabolite module was associated with subsequent CMV infection, eight metabolite modules were associated with conditioning regimens, and nine were associated with age upon aHSCT (Figure 1C). One lipid module was associated with sex (Figure 1D). All correlations between modules and clinical traits can be inspected in the interactive heatmaps in the Supplementary HTML file. The superpathway content for all the metabolite modules is shown in Figure 2A. The green metabolite module (containing 44 metabolites) that was inversely associated with CMV infection (Spearman’s Rank correlation = −0.36, q = 0.017) was found to contain seven amino acids, three cofactors and vitamins, four lipids, one peptide, eighteen xenobiotics, and eleven unknown metabolites (Figure 2B). These included multiple diet-derived molecules like TMAO (phospholipid metabolism), retinol and 4-oxo-retinoic acid (vitamin A metabolism), and ascorbic acid 3-sulfate (vitamin C biosynthesis) along with several metabolites related to benzoate metabolism or tryptophan metabolism. An interactive version of Figure 2B, along with corresponding figures for all the other modules, can be found in the Supplementary HTML file.

## 4. Discussion

In this study, we found that higher levels of trimethylamine N-oxide (TMAO) were associated with a lower risk of developing a subsequent CMV infection via a multivariable analysis. Using *WGCNA*, we were able to move from single-marker associations to a pathway-resolved, network-based analysis of metabolomics and lipidomics data. The analyses revealed that one module was associated with CMV infection and the type of conditioning regimen administered, while the other modules were associated with the administered conditioning regimen, age upon aHSCT, or sex. However, given the number of associations tested and the exploratory nature of the analyses, the biological and clinical relevance of these results should be interpreted cautiously, and the results require external validation.

The first aim of this study was to investigate metabolites previously associated with CMV infection and their associations with subsequent CMV infection in this cohort. We tested the associations of 12 metabolite measures, and only TMAO, which correlated inversely with CMV infection, was found to be associated. This result contradicts the previous findings acquired in the study by Monleón et al. 2015 [15], where higher levels of TMAO were associated with CMV infection in aHSCT patients after transplantation. These differences in results could be due to variations in the studies’ setups (Monleón et al. [15] measured TMAO post-CMV infection onset), the small cohort sizes (59 patients were included in the previous study, and 68 patients were included in our study), or unexplored confounding factors (e.g., haematological malignancy, diet, or other lifestyle-related variables). TMAO is formed from trimethylamine (TMA), which is produced by the gut microbiota following dietary intake of choline, phosphatidylcholine, and L-carnitine [24,25]. High levels of TMAO are associated with inflammation, involving, for instance, foam cell generation and vascular endothelium activation [24]. Therefore, one could expect high levels of TMAO in our cohort to be associated with CMV infection. This was, however, not the case. Given these contrasting results between our study and the literature, we can conclude that the complex relationship between CMV infections, conditioning regimens, diet, and metabolic profiles warrant further investigation. Further, we recommend that these further investigations are conducted using targeted metabolomics and lipidomics rather than the untargeted methods here.

The second aim of this study included an exploratory analysis of the patients’ metabolic and lipidomic profiles early post-aHSCT. We constructed modules consisting of 10 to 292 metabolites/lipids. The largest modules for both datasets were the grey modules, which are used to bin analytes that do not fit in any other module. The remaining modules are expected to contain molecules with shared biological properties or functions given their similar abundance profiles [21]. We tested the associations between the modules and subsequent CMV infection or other clinical traits known to be risk factors for CMV infection. We used *WGCNA* to link biological functions (in this case, metabolic pathways) to clinical phenotypes and outcomes. This method was originally designed for microarray and RNA-seq data but is fully applicable to other omics data with similar patterns of high correlation between analytes [26]. Using *WGCNA*, modules can be formed in a data-driven and unsupervised manner utilising the distance between molecules measured according to topological overlap. This allows for the assessment of both molecule–molecule correlations along with their shared correlations with other molecules in a dataset, constituting a method that represents the pathway structures of these types of data well. However, metabolites and lipids can have pleiotropic properties and can, therefore, potentially be placed equally well in multiple modules. This is not considered in *WGCNA*, as clustering is executed via non-overlapping partitioning. Further, this mode of partitioning is highly dependent on the selection of parameters, such as the correlation measure, the power, and the minimum module size. This is evident in our data, where we see a strong correlation between several module eigengenes in Figure S6, indicating that our specific partitioning may not be the most optimal form. Selecting parameters resulting in the best biological representation of data is a general and continuous issue. Therefore, resulting modules should always be assessed with caution, and further validation is essential.

The metabolic network analysis resulted in 16 modules, and the lipidomic analysis yielded 6 modules. When selecting the soft-threshold power for the lipidomics dataset, it was not possible to reach a scale-free topology model fit above 90% for powers ≤ 30 (i.e., the standard methodology for *WGCNA* [21]). This indicates that a subset of the samples in the lipidomics data exhibits a strong driving signal making it globally different from the remaining data, which would invalidate the assumption of a scale-free topology. Several of the modules correlated with either subsequent CMV infection, the conditioning regimen administered, sex, or age upon transplantation. Most associations were found with regard to conditioning regimen and age, reflecting that these factors have a large impact on the metabolism of aHSCT recipients. Further, the majority of the modules associated with conditioning regimens were also similarly associated with age, which is to be expected in this cohort, as the age of the transplant recipient is one of the determining factors whereby a conditioning regimen is selected (older patients are more likely to receive non-myeloablative conditioning, as they cannot withstand the myeloablative conditioning). One metabolite module, the green module, was inversely associated with subsequent CMV infection (higher levels of these molecules were associated with no CMV infection). Within this module, we found TMAO, which is in line with the results from our single-marker associations. The green module was also associated with the type of conditioning regimen employed and thus warrants further exploration in larger cohorts to better understand the impact of these metabolites on CMV infection and immune disruption caused by conditioning.

When performing system-level analyses using metabolomics and lipidomics data, it is important to acknowledge their limitations. First, despite advances in technologies, we are not able to detect all metabolites and lipids in biological samples [27]. Molecules have a wide variety of physicochemical properties and various baseline levels and are naturally fluctuating, all of which makes it difficult to detect them individually with high confidence. Thus, we might overlook relevant biological processes taking place within a patient cohort. Second, some molecules can be detected with spectrometry but are either unknown (209 (21%) of the metabolites in our dataset) or are not included in common libraries such as HMDB [28] (465 (48%) of the metabolites and 623 (63%) of the lipids in our datasets). This means that they cannot be included in standard annotation-based pathway enrichment analyses, which is why we chose a non-enrichment-based approach. Unknown molecules were kept in the analysis for their potential use in future studies, as metabolic libraries are continuously updated. Third, many of the biological functions carried out by molecules are not fully understood; therefore, we might overlook or misinterpret relevant biological signals. The detection methods used in this study are untargeted relative quantification metabolomics and untargeted fully quantitative lipidomics. Therefore, our metabolomics data cannot be directly compared to other datasets. Furthermore, the pathway annotations performed for the spectrometric data include only one super- and subpathway per molecule and thus do not provide a complete representation of biology, where the same molecule can take part in multiple functions and processes (pleiotropism). Finally, while this study has a larger sample size than previous studies [14,15], power was a limiting factor, particularly with respect to the correlation network analysis, where a large number of correlations were tested. Despite these limitations, we present these results as an explorative investigation of the post-aHSCT metabolic profiles in relation to clinical phenotypes that can be used to generate hypotheses for future studies. Further, the coding framework used to perform the analyses is readily available in easily approachable step-by-step scripts for use in other clinical studies.

## 5. Conclusions

In conclusion, we found an association between the dietary-derived metabolite TMAO and CMV infection occurring post-aHSCT. We were not able to confirm previous associations; this was likely due to potential confounding or false positive signals, the differences in the study designs, or the size of our cohort, which, although larger than previously explored cohorts, was still rather small. Using a pathway-resolved and network-based method, we were able to cluster metabolites and lipids based on their biological functions and further assess their relation to clinical traits related to CMV infection. Although exploratory, these analyses have identified clusters of metabolites that may be related to clinically relevant traits in this population, and these observations warrant further study with larger sample sizes. We further propose that correlation network analysis is a useful and hypothesis-free method for exploring the relationship between metabolite and lipid profiles in clinical populations and have provided fully documented, publicly available code to facilitate such research.

## Figures and Tables

**Table 1:** Cohort characteristics of cases (CMV-positive aHSCT recipients) and controls (CMV-negative aHSCT recipients). Values are presented as *n* (%) or median (min, max).

	All (*n* = 68)	Cases (*n* = 34)	Controls (*n* = 34)	*p*-Value
Male *	46 (72)	25 (74)	25 (71)	1 ^†^
Age upon aHSCT	56.5 (17, 73)	55.5 (22, 70)	59.5 (17, 73)	0.84 ^‡^
Sample collection in days post-aHSCT	28 (8, 33)	26.8 (14, 33)	28 (8, 33)	0.34 ^‡^
CMV infection onset in days post-aHSCT	48.5 (34, 90)	48.5 (34, 90)	-	
Graft origin				0.19 ^§^
Bone marrow	11 (16)	8 (24)	3 (9)	
Peripheral blood	57 (84)	26 (76)	31 (91)	
Conditioning regimen *				1 ^†^
Myeloablative conditioning (MAC)	28 (41)	14 (41)	14 (41)	
Non-myeloablative conditioning (Mini)	40 (59)	20 (59)	20 (59)	
CMV risk score **				0.00067 ^§^
Low	19 (28)	3 (9)	16 (47)	
Intermediate	21 (31)	11 (32)	10 (29)	
High	28 (41.2)	20 (58.8)	8 (24)	

* Used for matching cases and controls. ** Partially used for matching cases and controls. ^†^ Chi-square test; ^‡^ Wilcoxon independent rank-sum test, ^§^ Fisher’s exact test. aHSCT: allogeneic haematopoietic stem cell transplantation; CMV: cytomegalovirus; MAC: myeloablative conditioning. CMV risk score: Low = donor (D)−/recipient (R)− and D+/R−; Intermediate = D+/R+, High = D−/R+.

## Data Availability

The data are not publicly available as they were derived from patients treated in Denmark. The datasets contain sensitive patient data protected by GDPR and Danish law. Due to Danish legislation (Act No. 502 of 23 May 2018) and approvals granted by the Danish Data Protection Agency, it is not possible to upload raw data to a publicly available database. However, access to these data can be granted by the corresponding author upon reasonable request, provided a data transfer agreement is entered into that corresponds to current regulations.

## Supplementary Materials

The following supporting information can be downloaded at: https://www.mdpi.com/article/10.3390/metabo13090968/s1, Table S1: Sensitivity analysis of CMV risk groups. Figure S1: Distributions of data before and after log2 transformation. Table S2: Metabolites proposed to be associated with CMV infection in the literature. Figure S2: Power plots constructed with the *WGCNA* package. Figure S3: Method used for determining minimum module size parameters for the *WGCNA*. Table S3: Results from multivariable logistic regression of known associated metabolites. Figure S4: Collection times for the 68 samples included in the analysis. Table S4: Intermediate results of parameter selection for *WGCNA*. Table S5: Module names and sizes. Figure S5: Module membership (MM) distributions in modules resulting from the *WGCNA*. Figure S6: Correlations between modules resulting from the *WGCNA*. HTML: Results from *WGCNA*.
