# Supplementary material for: Metabolic Profiling Early Post-Allogeneic Haematopoietic Cell Transplantation in the Context of CMV Infection
Source: Metabolites. 2023 Aug 22;13(9):968. doi: 10.3390/metabo13090968 (PMC10536708; doi:10.3390/metabo13090968)
Supplement: Supplementary file 1 [file metabolites-13-00968-s001.zip › metabolites-2146362-supplementary.pdf]

Supplementary material

Table S1: Results from sensitivity analysis where low risk was used as reference to compare between group outcomes (CMV or no CMV infection). We found no difference between patients with unknown CMV risk and low CMV risk (p = 0.14). Therefore, the unknown CMV risk patients were grouped with the low-risk patients. Analysis performed using the glm function in R.

Term	Estimate	Std. error	*p*-value	95% CI
(Intercept)	1.10	0.07	0.15	0.97-1.26
Unknown risk	1.35	0.21	0.14	0.90-2.02
Intermediate risk	1.45	0.09	<0.01	1.22-1.73
High risk	1.93	0.09	<0.001	1.62-2.29

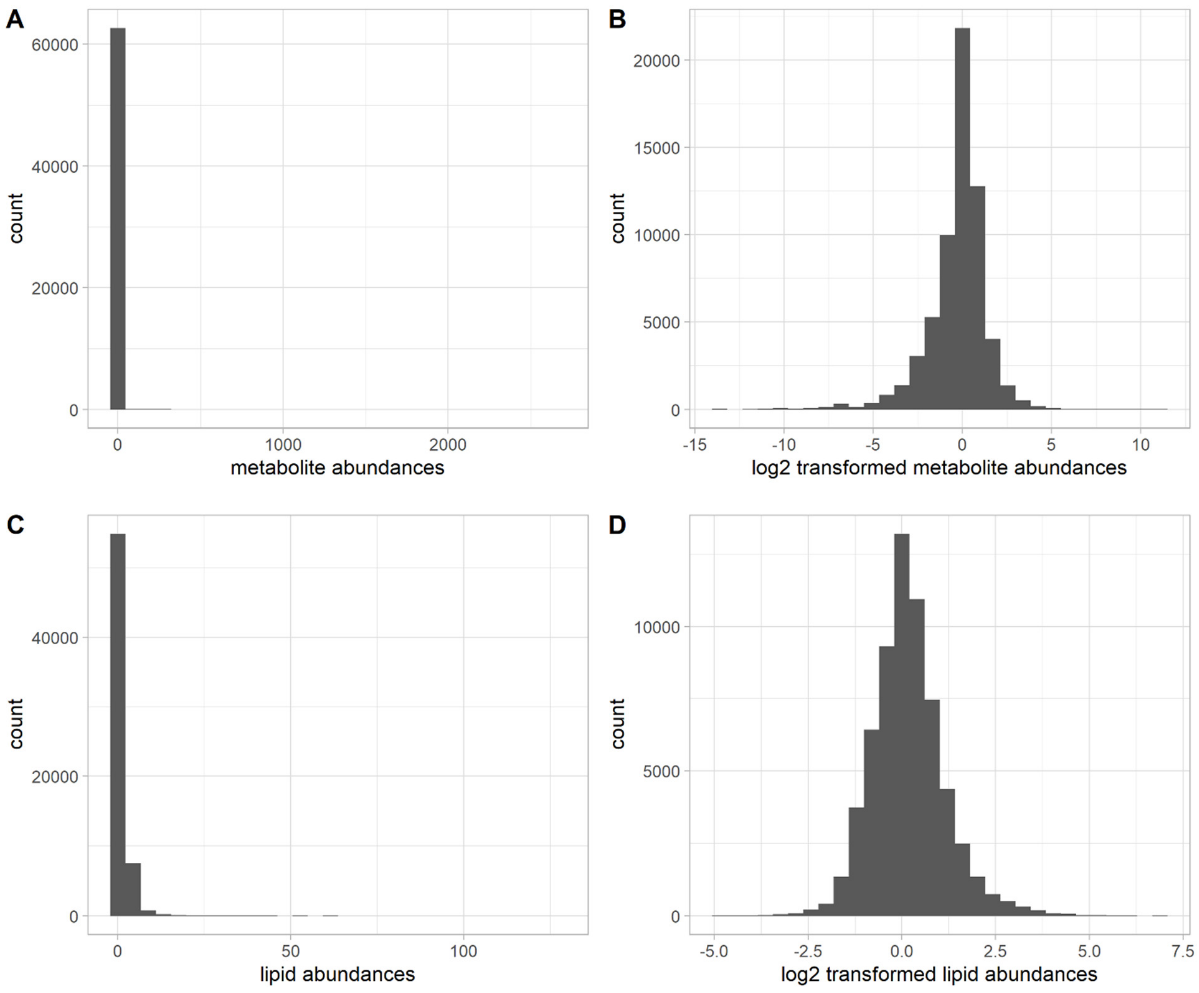

Figure S1: Distributions of data before and after log2 transformation. (**A**) Metabolite abundances before log2-transformation. (**B**) Metabolite abundances after log2-transformation. (**C**) Lipid abundances before log2-transformation. (**D**) Lipid abundances after log2-transformation.

Table S2: Metabolites proposed to be associated with CMV infection in previous studies. Description of relationship between metabolite(s) and CMV infection and which study reported the finding.

**Metabolite(s)**	**Association with CMV infection**	**Reference**
Glutamine	Required for viral replication in CMV infected human fibroblasts	Chambers et al., 2010
Phenylalanine, tryptophan	Positively linked to active CMV infection in kidney transplant patients	Sadeghi et al., 2011
Kynurenine, quinolinate	Positively linked to CMV disease severity in kidney transplant patients	Sadeghi et al., 2011
Alanine, total free fatty acids (FFA)	Positively associated with current CMV DNAemia in allogeneic haematopoietic stem cell transplantation (aHSCT) patients	Monleón et al., 2015
Choline, taurine, trimethylamine *N*-oxide (TMAO)	Positively associated with subsequent development of CMV DNAemia in aHSCT patients	Monleón et al., 2015
Lactate	Positively associated with current and subsequent development of CMV DNAemia in aHSCT patients	Monleón et al., 2015
Lysine	Negatively associated with current CMV DNAemia in aHSCT patients	Monleón et al., 2015

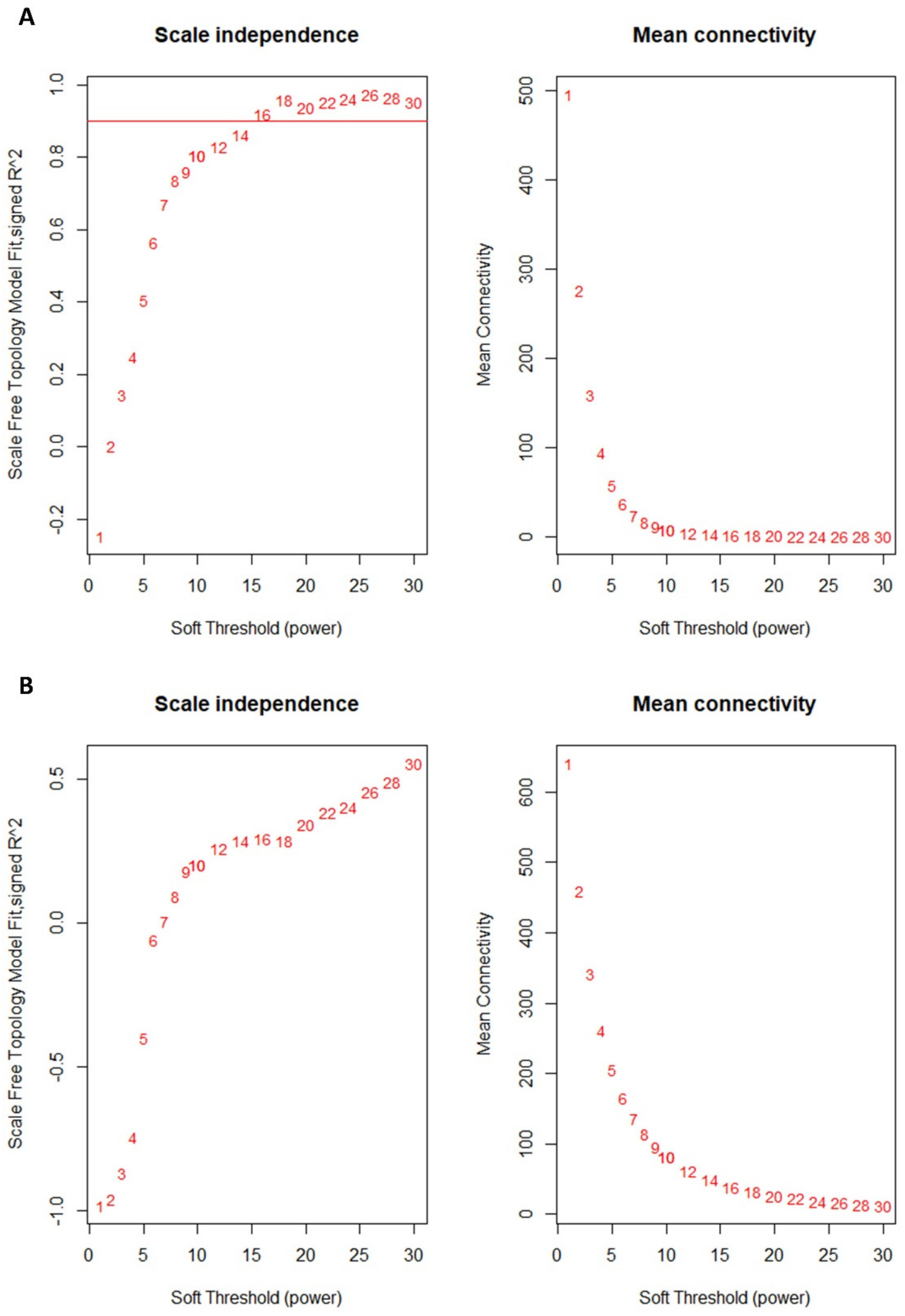

Figure S2: Power plots constructed with the *WGCNA* package. Scale independence plots (left panel) show the scale-free topology fit (y-axis) as a function of soft-threshold power values between 1-30 (x-axis). Mean connectivity plots (right panel) show the mean connectivity of the network (y-axis) as a function of soft-threshold power values between 1-30 (x-axis). (**A**) Metabolomics. A power of 16 was selected. (**B**) Lipidomics. A power of 30 was selected.

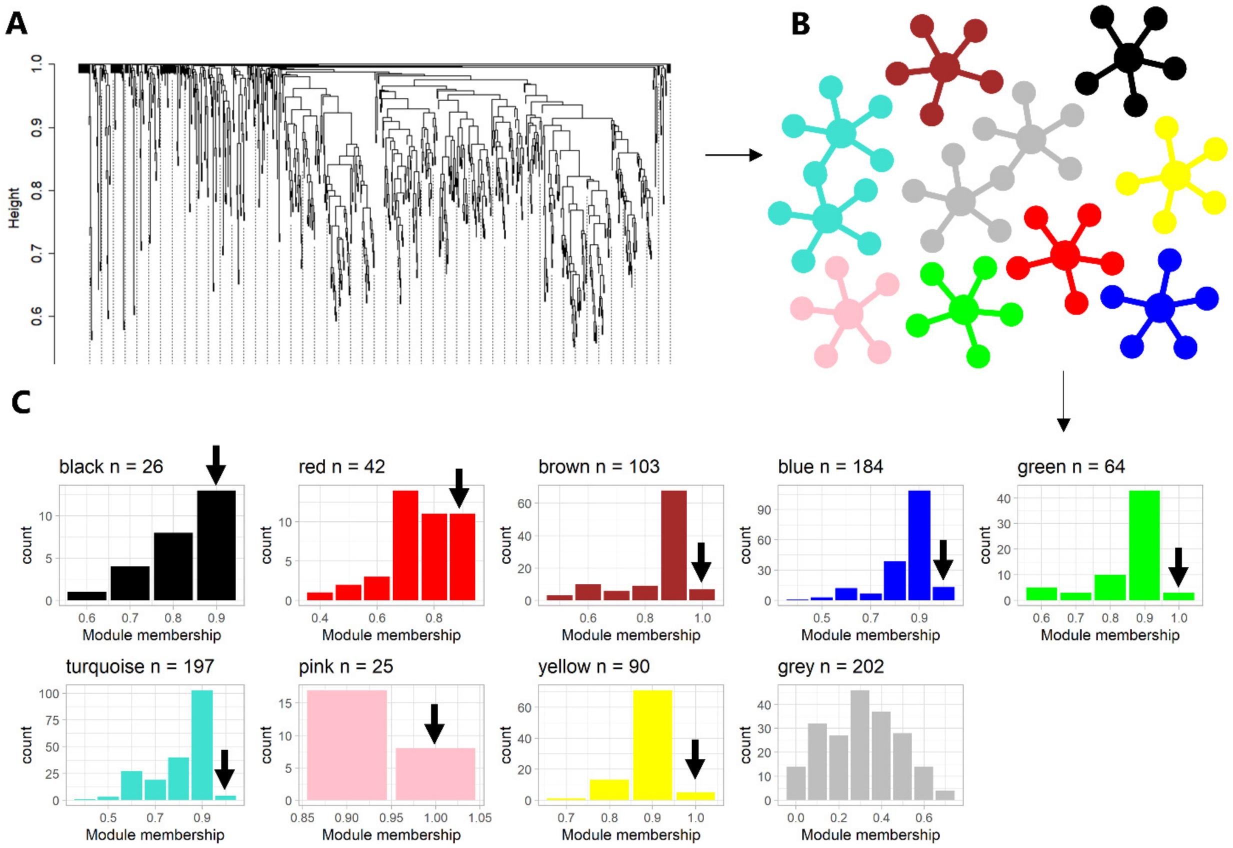

Figure S3: Schematic of method used for determining minimum module size parameter for the *WGCNA*. Steps B-C were performed for the minimum module sizes 5-20 with the aim of finding the optimal parameter value. (**A**) Clustering of molecules by topological overlap to create dendrogram. (**B**) Detect modules by cutting dendrogram. (**C**) Calculate module membership (MM) for each molecule (i.e., correlation between the molecule abundance profile and the first principal component of the module it was placed within). Find the highest module membership in each module and save the average across all modules (excluding the grey module which consists of metabolites/lipids not fitting in the remaining modules). Assess number of modules produced, their MM distributions, and the average highest MM for each value of minimum module size. The value resulting in a relatively small number of modules with high average max module membership and MM distributions skewed to the right was selected.

Table S3: Number of modules produced for each minimum module size value and the average max module membership (MM) for those modules. For the metabolomics dataset, a minimum module size of 10 was selected, and for the lipidomics dataset a value of 12 (highlighted in bold). These values were selected based on the numbers in this table and the distribution of module memberships (distributions for final selected modules can be found in Figure S5, distributions for modules formed with other minimum module size values are not shown).

**Metabolomics**			**Lipidomics**		
Minimum module size	Number of modules	Average max MM	Minimum module size	Number of modules	Average max MM
5	33	0.92	5	19	0.96
6	29	0.92	6	12	0.96
7	24	0.92	7	11	0.96
8	20	0.92	8	9	0.98
9	16	0.92	9	9	0.98

**10**	**16**	**0.92**	10	9	0.98
11	15	0.91	11	9	0.98
12	12	0.91	**12**	**9**	**0.98**
13	12	0.91	13	9	0.96
14	12	0.91	14	9	0.96
15	11	0.90	15	9	0.96
16	11	0.90	16	9	0.96
17	10	0.89	17	9	0.96
18	8	0.90	18	9	0.96
19	6	0.90	19	9	0.96
20	6	0.90	20	9	0.98

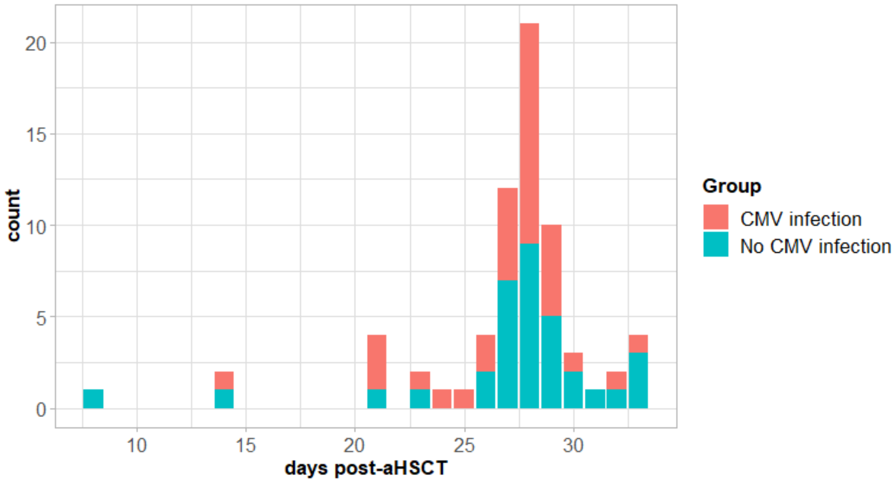

Figure S4: Collection times for the 68 samples included in the analysis. All samples were collected between days 8-33 post-allogeneic haematopoietic stem cell transplantation (aHSCT). Patients from the *CMV infection* group all tested positive for CMV infection after sample collection and between days 34-100 post-aHSCT. Patients from the *No CMV infection* group had no positive CMV PCR test within 100 days post-aHSCT.

Table S4: Results from multivariable logistic regression of downstream CMV infection and metabolites previously associated with CMV infection. Models were adjusted for sex, age at aHSCT, conditioning regimen, and CMV risk score. *P*-values have not been adjusted for multiple testing. Significance threshold $p < 0.05$, significant results highlighted in bold.

**Metabolite**	**OR**	**95% CI**	***p*-value**
Alanine	1.12	0.58-3.50	0.76
Choline	1.55	0.08-31.7	0.77
Glutamine	9.78	0.39-402	0.19
Kynurenine	2.07	0.57-8.40	0.28
Lactate	0.46	0.11-1.78	0.27
Lysine	1.22	0.21-11.1	0.83
Phenylalanine	3.39	0.66-54.0	0.33
Quinolinate	1.33	0.88-2.12	0.20
Taurine	0.67	0.24-1.82	0.41

Total FFA	1.12	0.18-5.96	0.89
**Trimethylamine *N*-oxide**	**0.63**	**0.41-0.87**	**0.01**
Tryptophan	1.39	0.32-11.3	0.69

Table S5: Modules constructed using *WGCNA* and the number of molecules placed within each. The grey module is used as bin for molecules that do not fit in the remaining modules.

**Metabolite modules, n = 922**	**Lipid modules, n = 933**
Midnightblue, n = 10	Pink, n = 25
Cyan, n = 11	Black, n = 26
Salmon, n = 11	Red, n = 42
Tan, n = 13	Green, n = 64
Greenyellow, n = 19	Yellow, n = 90
Purple, n = 20	Brown, n = 103
Magenta, n = 24	Blue, n = 184
Pink, n = 31	Turquoise, n = 197
Black, n = 32	Grey, n = 202
Red, n = 38	
Green, n = 44	
Yellow, n = 51	
Blue, n = 62	
Brown, n = 62	
Turquoise, n = 202	
Grey, n = 292	

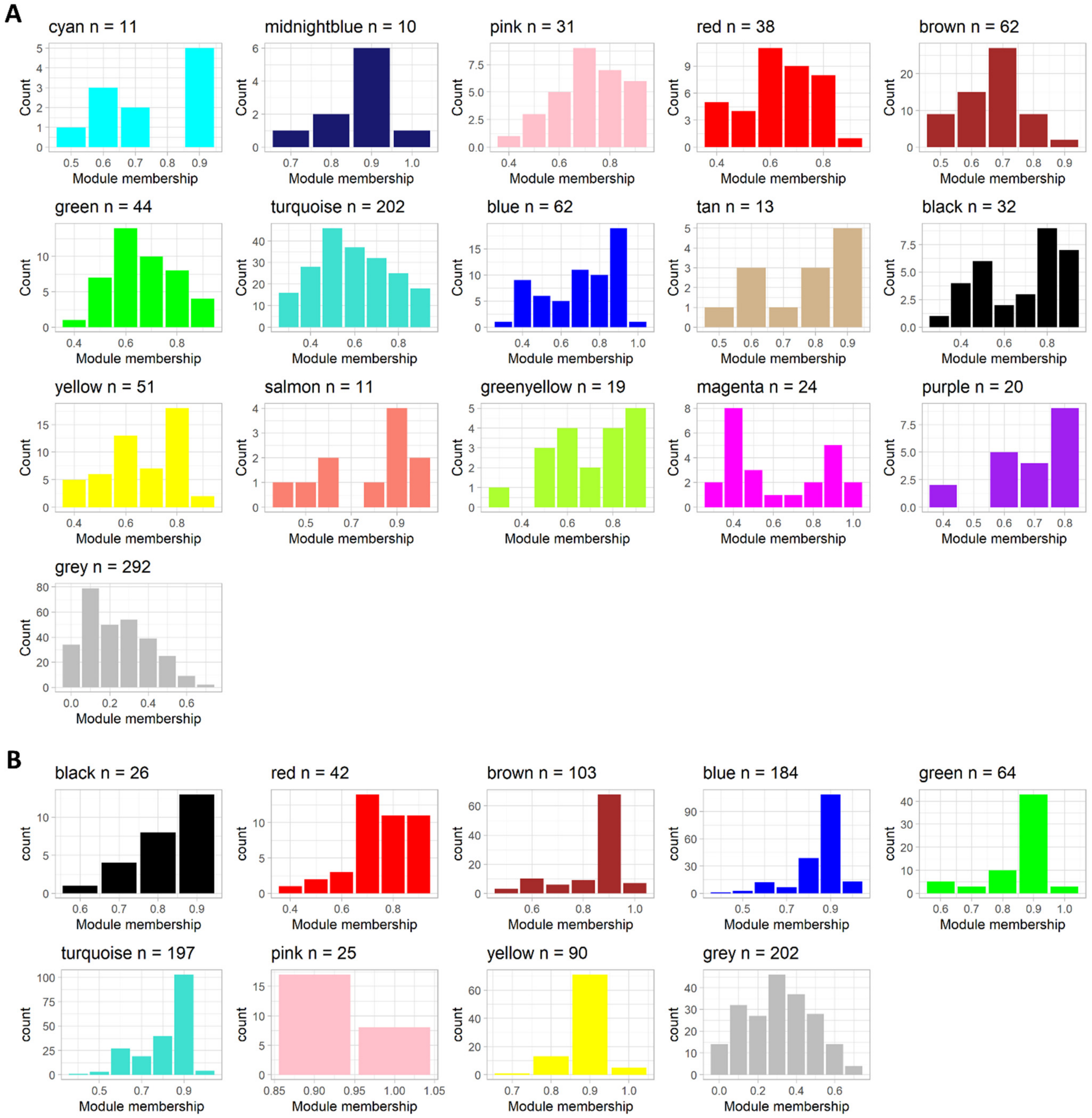

Figure S5: Module membership (MM) distributions in modules, defined by a random colour, resulting from the *WGCNA*. Same colour modules across datasets are not related. Module memberships are calculated as the correlation between the metabolite abundance profile and the first principal component of the assigned module. Each subplot represents a module, x-axis the module membership, and y-axis the number of metabolites with that module membership. (**A**) Metabolite modules constructed with a minimum module size of 10. (**B**) Lipid modules produced with a minimum module size of 12.

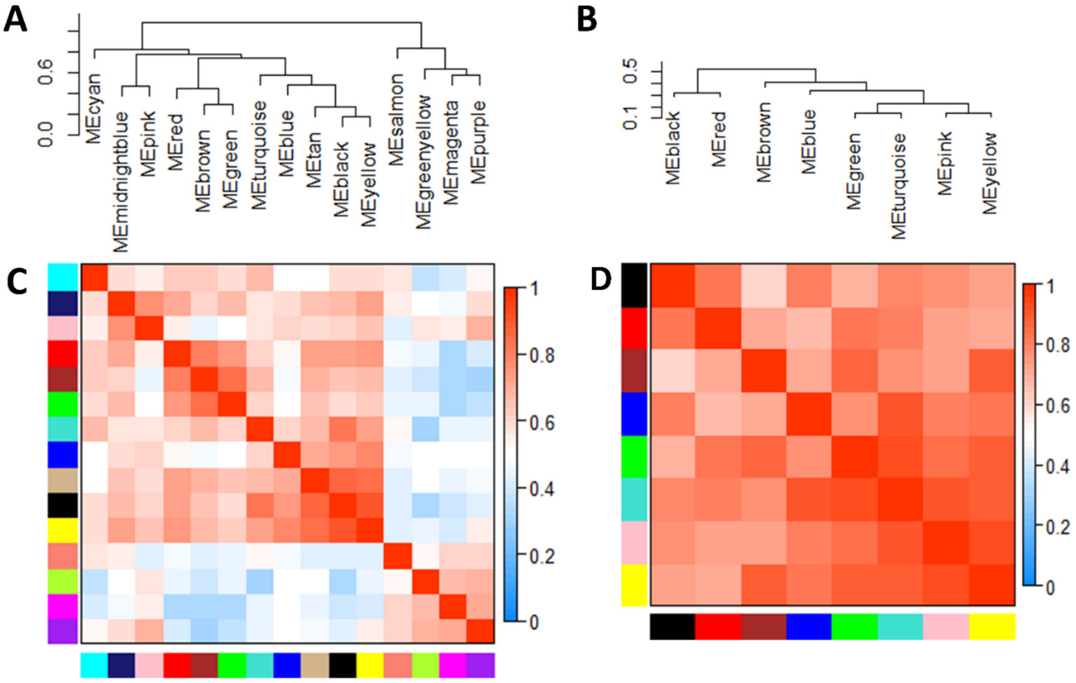

Figure S6: Correlations between modules resulting from the *WGCNA*. (**A+B**) Dendrograms showing a hierarchical clustering of the metabolite module eigengenes (MEs) (**A**) and the lipid MEs (**B**). (**C+D**) Absolute Spearman's rank correlation heatmap of metabolite MEs (**C**) and lipid MEs (**D**). The correlation coefficient is indicated by the colour bar to the right of each heatmap, blue being no correlation and red being high correlation.
