# Supplementary material for: Metabolic Profiling Early Post-Allogeneic Haematopoietic Cell Transplantation in the Context of CMV Infection
Source: Metabolites. 2023 Aug 22;13(9):968. doi: 10.3390/metabo13090968 (PMC10536708; doi:10.3390/metabo13090968)
Supplement: Supplementary file 1 [file metabolites-13-00968-s001.zip › WGCNA_module_results.html]

Supplemental figures and tables


# Supplemental figures and tables

### *Metabolic profiling early post allogeneic haematopoietic cell transplantation in the context of CMV infection*

#### By Kirstine K. Rasmussen

#### 14 december, 2022

- Table of molecules and
  modules
- Module memberships
- Module
  correlations with clinical traits
- Super-pathway content in
  modules
- Sub-pathway content per
  module
- Descriptive statistics

The following tables and figures are the result of a weighted
correlation network analysis of metabolomics and lipidomics data from
plasma samples of patients who underwent an allogeneic haematopoietic
stem cell transplantation at Rigshospitalet, Copenhagen University
Hospital, Denmark in the period January 2016 to October 2017. The
analyses were performed using the *Weighed Gene Co-expression Network
Analysis* (WGCNA) R package (link)
which identifies modules (clusters) of analytes with high topological
overlap. The metabolomics and lipidomics data were analysed separately
and clusters between datasets are not associated. The figures shown in
this document were constructed to visualise the results from the WGCNA
and are not part of the package.

The content of this file is presented to foster hypothesis-generation
for use in future studies. All code is available in GitHub (link).

---

## Table of molecules and modules

All metabolites and lipids included in the analyses are found in the
tables below. You can sort or search by module to get all molecules in a
single module, or by pathway to see how they are distributed across
modules. Metabolites with names ‘X - #####’ are those detected by use of
HPLC-MS/MS but not annotated in the reference library.

### Metabolites

### Lipids

---

## Module memberships

The figures below show the distribution of module memberships
(definition below) in each module. The title in each subplot describes
which module is represented and the amount of molecules placed within
it. The grey module is in WGCNA used as a bin for molecules not fitting
in the remaining modules.

*Module membership:* A term used in the WGCNA package to
describe the correlation between a molecule abundance profile and the
first principal component (weighted average) of all molecules in the
same module.

### Metabolites

### Lipids

---

## Module correlations with clinical traits

The figures below show correlation heatmaps of modules and clinical
traits. Each module was tested for correlation with a selection of
clinical variables related to the risk of CMV infection. Correlations
were calculated with the Spearman’s Rank correlation measure and
evaluated by Fisher’s exact statistics and FDR adjusted p-values
(q-values). You can hover over the cells to see exact correlations and
q-values.

### Metabolites

### Lipids

*q value significance:* \*<0.05, \*\*<0.01,
\*\*\*<0.0001.

*CMV infection:* Detected CMV infection within 100 days
post-aHSCT; 0 = no detected CMV, 1 = detected CMV.

---

## Super-pathway content in modules

The figures below shows the super-pathway content per module.
Super-pathways are represented on the x-axis by color-coded bars. The
percentage of molecules annotated to each super-pathway placed in each
module is indicated on the y-axis. E.g. 10% of all energy related
metabolites are placed within the black module.

### Metabolites

### Lipids

---

## Sub-pathway content per module

Metabolic pathway content within each module is presented below in
donutplots. Click between tabs to view each module. Some modules
contains too many molecules from the same sub-pathway to be shown in
full. Complete lists can be found by searching the modules in the tables
above.

### Metabolites

#### blue

#### turquoise

#### yellow

#### red

#### green

#### grey

#### purple

#### brown

#### salmon

#### tan

#### pink

#### magenta

#### black

#### greenyellow

#### cyan

#### midnightblue

### Lipids

#### blue

#### grey

#### red

#### turquoise

#### brown

#### green

#### yellow

#### black

#### pink

## Descriptive statistics

Basic descriptive statistics for the complete cohort, the cases, and
the controls.

### Metabolites

#### All cohort

#### CMV positive cases

#### CMV negative controls

### Lipids

#### All cohort

#### CMV positive cases

#### CMV negative controls
